# High Throughput Molecular Characterization of Normal Karyotype Acute Myeloid Leukemia in the Context of the Prospective Trial 02/06 of the Northern Italy Leukemia Group (NILG)

**DOI:** 10.3390/cancers12082242

**Published:** 2020-08-11

**Authors:** Silvia Salmoiraghi, Roberta Cavagna, Pamela Zanghì, Chiara Pavoni, Anna Michelato, Ksenija Buklijas, Lara Elidi, Tamara Intermesoli, Federico Lussana, Elena Oldani, Chiara Caprioli, Paola Stefanoni, Giacomo Gianfaldoni, Ernesta Audisio, Elisabetta Terruzzi, Lorella De Paoli, Erika Borlenghi, Irene Cavattoni, Daniele Mattei, Annamaria Scattolin, Monica Tajana, Fabio Ciceri, Elisabetta Todisco, Leonardo Campiotti, Paolo Corradini, Nicola Fracchiolla, Renato Bassan, Alessandro Rambaldi, Orietta Spinelli

**Affiliations:** 1 Hematology Unit, Azienda Socio Sanitaria Territoriale (ASST), Ospedale Papa Giovanni XXIII, 24127 Bergamo, Italy; ssalmoiraghi@fondazionefrom.it (S.S.); roberta.cavagna@unimi.it (R.C.); pamzangh@libero.it (P.Z.); cpavoni@asst-pg23.it (C.P.); amichelato@asst-pg23.it (A.M.); ksenija.buklijas@gmail.com (K.B.); lara.elidi@icloud.com (L.E.); tintermesoli@asst-pg23.it (T.I.); flussana@asst-pg23.it (F.L.); eoldani@asst-pg23.it (E.O.); chiara.caprioli@unimi.it (C.C.); pstefanoni@asst-pg23.it (P.S.); ospinelli@asst-pg23.it (O.S.); 2 FROM Research Foundation, Papa Giovanni XXIII Hospital, 24127 Bergamo, Italy; 3 PhD Program in Translational and Molecular Medicine, University of Milano-Bicocca, 20126 Milano, Italy; 4 Hematology Unit, Azienda Ospedaliera Universitaria Careggi, 50134 Firenze, Italy; ggianfaldoni@libero.it; 5 Hematology Unit A.O.U. Città della Salute e della Scienza di Torino, 10126 Torino, Italy; eaudisio@cittadellasalute.to.it; 6 Hematology Unit, Azienda Ospedaliera San Gerardo, 20900 Monza, Italy; eterruzzi@yahoo.com; 7 Hematology Unit, Azienda Ospedaliera SS. Antonio e Biagio e Cesare Arrigo, 15121 Alessandria, Italy; ldepaoli@ospedale.al.it; 8 Hematology Unit, ASST-Spedali Civili, 25123, Brescia, Italy; erika.borlenghi@gmail.com; 9 Hematology Unit, Ospedale S. Maurizio, 39100 Bolzano, Italy; irenemaria.cavattoni@sabes.it; 10 Hematology Unit, Azienda Ospedaliera S.Croce e Carle di Cuneo, 12100 Cuneo, Italy; mattei.d@ospedale.cuneo.it; 11 Hematology Unit, Ospedale dell’Angelo and SS. Giovanni e Paolo, 30174 Venezia Mestre, Italy; annamaria.scattolin@aulss3.veneto.it (A.S.); renato.bassan@aulss3.veneto.it (R.B.); 12 Hematology Unit, Azienda Socio Sanitaria Territoriale (ASST) Ospedale di Cremona, 26100 Cremona, Italy; mtajana@tiscali.it; 13 Hematology Unit, IRCSS Ospedale San Raffaele, 20132 Milano, Italy; ciceri.fabio@hsr.it; 14 Hematology Unit, IRCCS Istituto Clinico Humanitas di Rozzano, 20089 Rozzano (MI), Italy; elisabetta.todisco@ieo.it; 15 Medicine and Surgery Department, University of Insubria, 21100 Varese, Italy; leonardo.campiotti@uninsubria.it; 16 Hematology Unit, Fondazione IRCCS Istituto Nazionale dei Tumori, 20133 Milano, Italy; paolo.corradini@unimi.it; 17 Oncology and Hematoncology Department, University of Milan, 20122 Milano, Italy; 18 Hematology Unit, Fondazione IRCCS Ca’ Granda Ospedale Maggiore Policlinico, 20122 Milano, Italy; nicola.fracchiolla@policlinico.mi.it

**Keywords:** Acute Myeloid Leukemia, molecular marker, NGS

## Abstract

By way of a Next-Generation Sequencing NGS high throughput approach, we defined the mutational profile in a cohort of 221 normal karyotype acute myeloid leukemia (NK-AML) enrolled into a prospective randomized clinical trial, designed to evaluate an intensified chemotherapy program for remission induction. *NPM1*, *DNMT3A,* and *FLT3*-ITD were the most frequently mutated genes while *DNMT3A*, *FLT3*, *IDH1*, *PTPN11*, and *RAD21* mutations were more common in the *NPM1* mutated patients (*p* < 0.05). *IDH1* R132H mutation was strictly associated with *NPM1* mutation and mutually exclusive with *RUNX1* and *ASXL1*. In the whole cohort of NK-AML, no matter the induction chemotherapy used, by multivariate analysis, the achievement of complete remission was negatively affected by the *SRSF2* mutation. Alterations of *FLT3* (*FLT3-*ITD) and *U2AF1* were associated with a worse overall and disease-free survival (*p* < 0.05). *FLT3-*ITD positive patients who proceeded to alloHSCT had a survival probability similar to *FLT3-*ITD negative patients and the transplant outcome was no different when comparing high and low-AR-*FLT3*-ITD subgroups in terms of both OS and DFS. In conclusion, a comprehensive molecular profile for NK-AML allows for the identification of genetic lesions associated to different clinical outcomes and the selection of the most appropriate and effective treatment strategies, including stem cell transplantation and targeted therapies.

## 1. Introduction

Cytogenetic analysis has proved to be crucial for the prognostic stratification of acute myeloid leukemia (AML) patients [1]. However, nearly half of AML patients have a normal karyotype (NK). The identification of molecular mutations has dramatically improved our knowledge of AML molecular genetics and shed new light not only on the molecular pathogenesis of the disease but also on the prognostic significance of each mutation and their combination in NK-AML [2,3]. *NPM1* mutations are found in approximately one third of AML and in about 50% of cases with a normal karyotype [1,4,5]. Alterations involving *NPM1* often occur in combination with other genetic aberrations, which may contribute to determining the disease evolution [3]. Moreover, about 30% of NK-AML [6] is affected by *FLT3*-internal tandem duplication (ITD) resulting in the deregulation of *flt3* kinase activity and determining a worse clinical outcome, even in the presence of *NPM1* mutations [7,8]. Particularly, the evaluation of the *FLT3* allelic ratio (AR) has been included in the European leukemia net (ELN) classification to further improve risk stratification in *FLT3*-ITD mutated AML patients [1], even if this remains a matter of debate [9]. The molecular characterization of AML, obtained by the application of high throughput sequencing, has led to a better classification of this disease and its prognostic profile [1,10]. However, most NK-AML belong to the broad intermediate prognostic subgroup in which the most appropriate treatment strategy remains to be defined. This seems particularly relevant when considering the new drugs targeting specific mutations [11] and the benefit potentially gained by allogeneic transplantation as post remission consolidation treatment in these patients.

In this context, the purpose of this study was to define the association of molecular mutations with the outcome of a cohort of 221 NK-AML patients treated according to a prospective trial comparing a standard vs. high-dose chemotherapy regimen for remission induction (ClinicalTrials.gov identifier: NCT00495287) [12].

## 2. Results

### 2.1. Clinical and Molecular Findings

The clinical characteristics of the 221 NK-AML patients included in this analysis are summarized in Table 1. The median age at diagnosis was 52 years (range, 19–74 years) and the majority of them (88%) had a de novo AML. The clinical and biological patient characteristics were generally well balanced between the induction arms of the study (Table 1).

**Figure 1:**
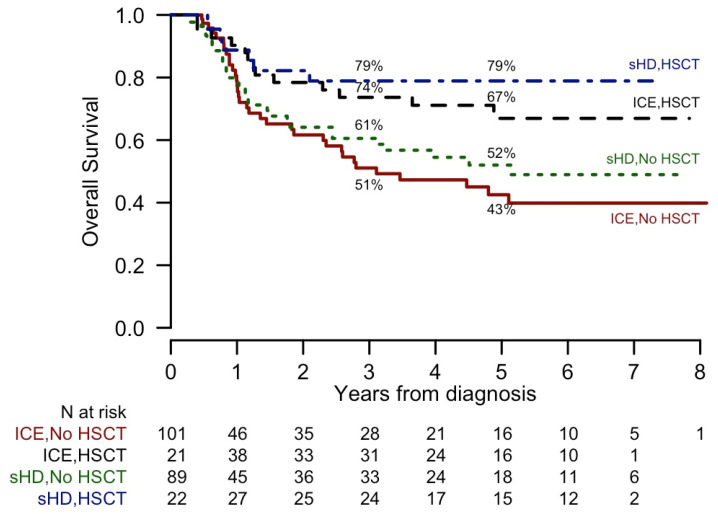
Kaplan-Meier curves of Overall Survival (OS), according to induction and consolidation treatments, in complete remission patients. 5-year OS estimates are reported. *p* values assessed comparing groups are: HSCT, sHD vs. ICE: *p* = 0.48; No HSCT, sHD vs. ICE: *p* = 0.52; sHD, HSCT vs. no HSCT: *p* = 0.03; ICE, HSCT vs. no HSCT: *p* = 0.01.

According to trial indications [12], 71 out of 190 molecular profiled patients in first complete remission (CR) underwent alloHSCT (Figure 1).

**Figure 2:**
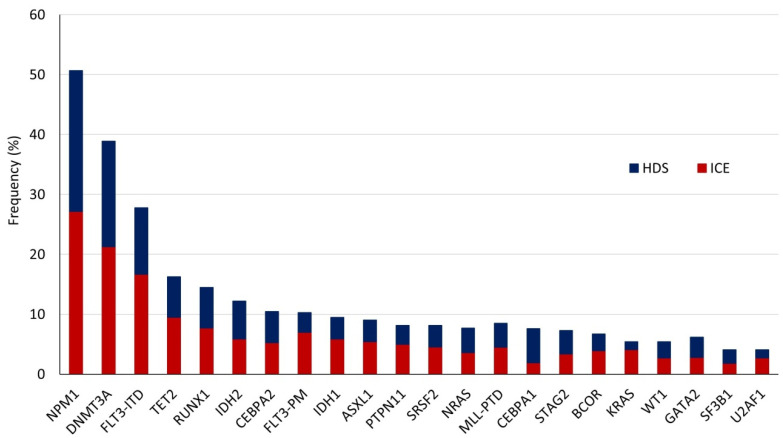
Frequency of different mutated genes according to induction treatment. *CEBPA*2 and *CEBPA*1 indicate the presence of double or single mutation, respectively.

**Figure 3:**
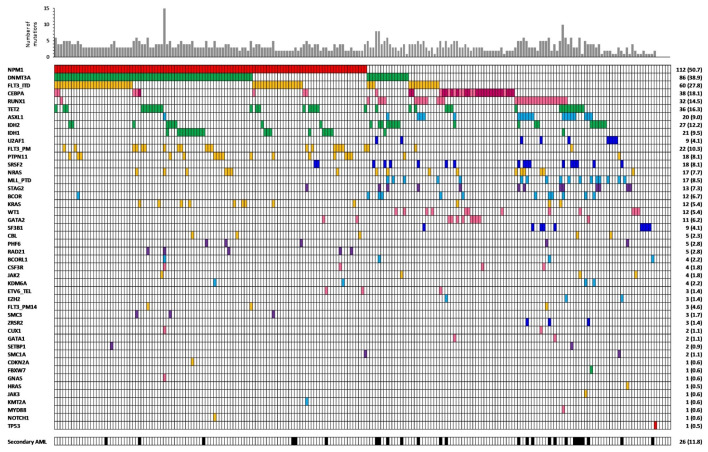
Frequency of different mutated genes in our cohort of patients. In *CEBPA* line, dark pink indicates the presence of a double mutation.

**Figure 4:**
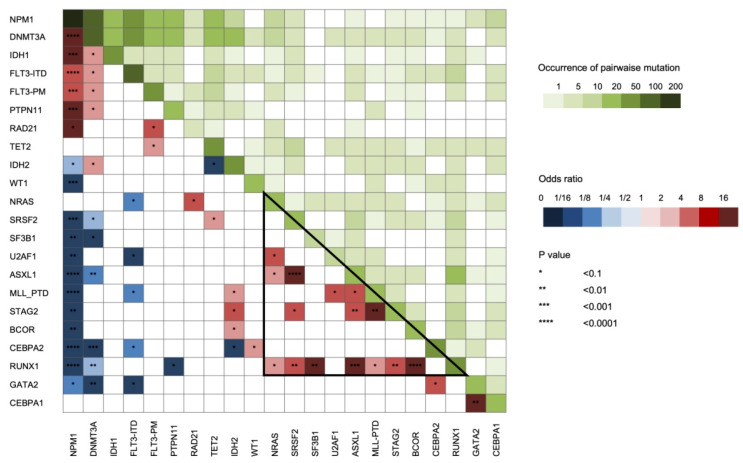
Pairwise association among gene mutations. The odds ratio of the association is color coded: blue colors indicate a negative association while red colors indicate a positive association. In addition, differential green intensity represent a different co-occurrence of mutations in terms of number of patients. Triangle indicates a group of genes which frequently co-mutate in *NPM1* wild-type AML. *CEBPA*2 and *CEBPA*1 indicate the presence of double or single mutation, respectively.

The NGS analysis of the 221 patients identified a total of 738 mutations, including non-synonymous point mutations (missense (*n* = 334) and nonsense (*n* = 42)), insertions or deletions (indels) (in frame (*n* = 112) or causing a frameshift (*n* = 226)), and splicing sites mutations (*n* = 24). The number of molecular alterations per patient ranged from 0 to a maximum of 15, with a median of 3. Only five patients did not present mutations detectable by the applied gene panel. The mutation frequencies according to induction treatment are reported in Figure 2, whereas the number of alterations per patient and per gene are represented in Figure 3. Moreover, we measured the association between mutations in different genes, considering genes in pairs (Figure 4).

As expected, the most frequently mutated gene in our cohort of patients was *NPM1*, followed by *DNMT3A* and *FLT3*. We noticed that *DNMT3A*, *FLT3*, *IDH1*, *PTPN11*, and *RAD21* mutations were more common in the *NPM1* mutated patients (*p* < 0.05). In particular, *IDH1* R132H mutation was strictly associated with *NPM1* mutation and mutually exclusive with *RUNX1* and *ASXL1* while the R132C was not [13]. Alterations involving the *IDH2* gene in specific amino-acids showed a different behavior regarding co-occurrence with other genes lesions. Particularly, *IDH2* R140 mutation was associated with the presence of *NPM1* alteration and rarely with *RUNX1* mutations, while the amino-acid changes involving R172 presented the opposite combinations [14]. As expected, *RUNX1* mutations often co-occurred with alterations in *ASXL1*, *BCOR*, *SF3B1*, *SRSF2*, *STAG2*, *NRAS*, and *KMT2A*-PTD [15], and within this latter group of genes, pathologic variants were also frequently present in combination (Figure 4). *BCOR* mutations were virtually mutually exclusive with *NPM1* mutations while associated with *RUNX1* alterations [16]. Lastly, *TP53* mutations were revealed only in one NK-AML patient as solely identified genetic aberration (Figure 3). Interestingly, this patient harbored two point mutations probably affecting two different alleles, as commonly described for tumor suppressor genes.

### 2.2. Impact of Clinical and Molecular Profiling on CR Achievement

By univariate analysis, (Table 2) age, Eastern Cooperative Oncology Group (ECOG) performance status (PS), de novo AML nature and gene mutation profile at diagnosis had an impact on CR achievement.

In particular, achievement of CR was negatively affected by the presence of molecular alterations in *TET2*, *ASXL1* and *SRSF2* genes. On the contrary, the group of patients characterized by the presence of an *NPM1* gene mutation in the absence of *FLT3-*ITD showed a significantly higher probability to achieve CR (Table 2). The presence of a double mutation in *CEBPA* gene was associated with a favorable hazard ratio (HR) for CR achievement. By multivariate analysis, the negative effect of the presence of an altered *SRSF2* gene on CR achievement was confirmed (Table 3).

The probability to reach CR was not different according to the treatment allocation when a forest plot analysis was applied to each mutation (Figure S1).

### 2.3. Impact of Clinical and Molecular Characteristics on Survival

**Figure 5:**
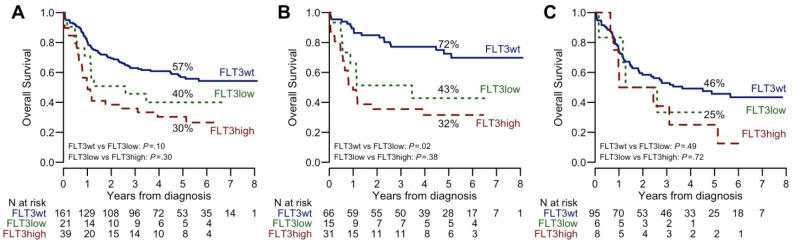
Kaplan-Meier curves of Overall Survival (OS), according to *FLT3*-ITD ratio. (**A**) All patients; (**B**) *NPM1* positive patients; (**C**) *NPM1* wild-type patients. 5-year OS estimates and *p* values are reported.

Survival analysis showed that age, ECOG PS and white blood counts influenced the clinical outcome of NK-AML (Table 2). Mutations of *FLT3 (FLT3-ITD)*, *RUNX1*, and *U2AF1* were associated with a worse OS and DFS (*p* < 0.05) while double alterations involving *CEBPA* gene proved to have a favorable impact on clinical outcome, both in terms of OS and DFS (*p* < 0.05). Patients with *NPM1* gene mutations but negative for *FLT3-ITD* had a better OS and DFS (*p* = 0.0001 and 0.0009, respectively) (Table 2 and Figure S2). This survival advantage was particularly evident in patients randomized to high-dose chemotherapy during the induction phase (Figure S3). Conversely, the presence of *NPM1* gene mutations did not improve the clinical outcome of patients also bearing *FLT3-ITD* alteration. A gradient effect on survival was documented when *FLT3-ITD* positive patients were classified according to ELN guidelines 2017 as low-AR-*FLT3*-ITD (allelic ratio, AR < 0.5) or high-AR (AR ≥ 0.5) (Table 2, Figure 5).

We also verified if the allelic burden calculated for *NPM1* mutations (variant allelic fraction, VAF ≤ 0.4 or > 0.4) could have an impact on outcome as recently reported [17] but we did not observe any correlation between *NPM1* VAF and clinical outcome in our cohort of patients. By multivariate analysis (Table 3), the positive effect on survival of an aberrant *NPM1* and a double mutated *CEBPA* was confirmed. In addition, the negative effect on survival related to *FLT3*-ITD as well as mutations involving *U2AF1* gene remained statistically significant also by multivariate analysis.

The univariate analysis showed that the presence of *FLT3*-ITD abolished the prognostic impact of any other identified mutation. By contrast, in patients with no *NPM1* or *FLT3-ITD* mutations, the presence of *DNMT3A*, *TET2*, *RUNX1*, *NRAS,* and *U2AF1* negatively affected survival (Table 4).

In this subgroup, the unfavorable prognostic effect of *U2AF1* mutations on survival remained significant also by multivariate analysis. The presence of a *RUNX1* mutation was associated with an unfavorable, despite not statistically significant, HR for survival (Table 5).

### 2.4. Impact of alloHSCT by Molecular Lesions

**Figure 6:**
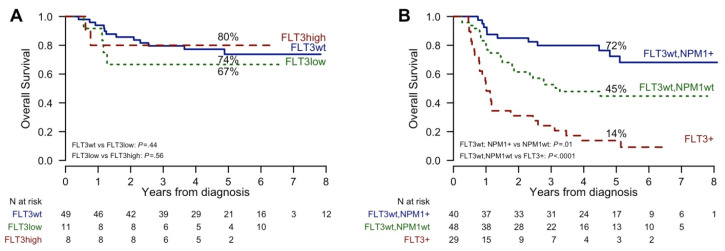
Kaplan-Meier curves of Overall Survival (OS) in different consolidation programs. (**A**) Patients receiving allogeneic stem cell transplantation, according to *FLT3*-ITD ratio; (**B**) Patients receiving other consolidation program, according to *FLT3*-ITD and *NPM1* mutations. 5-year OS estimates and *p* values are reported.

The 22 *FLT3-ITD* positive patients who could proceed to alloHSCT had a survival probability similar to *FLT3-ITD* negative patients. The transplant outcome was not different when comparing high and low-AR-*FLT3*-ITD subgroups both in terms of OS (Figure 6) and DFS. The OS of *FLT3*-ITD positive patients, no matter if *NPM1* negative or positive, who did not receive alloHSCT for whatever reason showed quite a poor outcome (Figure 6B, *p* < 0.00001). The limited number of patients precluded the possibility to evaluate the ability of alloHSCT to modify the adverse outcome associated with other molecular alterations.

## 3. Discussion

In this study, we provide an accurate molecular characterization of 221 NK-AML patients included in a prospective clinical trial comparing the standard ICE induction chemotherapy to the high-dose regimen. By applying an NGS high throughput solution to sequence myeloid neoplasms related genes, we were able to identify at least one mutation in the great majority of patients (98%). Frequencies and co-occurrence of mutations are consistent with previous observations [3,14]. Our data confirm that the identification of *CEBPA*, *NPM1*, and *FLT3-ITD* mutations, alone or in combination, remains crucial to define patient subgroups with different prognoses. Double mutation in *CEBPA* gene identifies a subgroup of patient characterized by a particularly favorable outcome. On the contrary, *FLT3-*ITD mutations represent the most relevant marker of unfavorable prognosis in this setting, no matter the presence of *NPM1* or other gene mutations. We observed a gradient effect played by *FLT3*-ITD allele burden on survival since, the low-AR-*FLT3*-ITD was still associated to a negative outcome. This correlation was not statistically significant probably due to the sample size of low-AR-FLT3-ITD subgroups (with or without mutant *NPM1*) which is relatively low. This observation is in line with other studies [9,18] and represents an open challenge as to the choice of the post-remission strategies. Within the limit of a modest number of patients so far analyzed, our results suggest that alloHSCT can abolish the adverse effect due to the *FLT3*-ITD mutation. For these reasons, at our institution, alloHSCT remains the preferred post remission option for patients with low-AR-*FLT3*-ITD. The role of innovative *FLT3* inhibitors, either to improve the transplant outcome or to avoid it, will perhaps modify the therapeutic scenario of this AML subgroup [19,20,21]. The *FLT3*-ITD mutation exerts its negative influence also in *NPM1* mutated patients. This observation supports the paradigm of how the presence of co-occurring mutations can modify the effect of a single mutation on the prognosis [22] and demonstrates the importance of refining molecular characterization of AML at disease presentation.

In patients with no mutations of both *FLT3*-ITD and *NPM1*, additional mutations in other leukemia-related genes proved to influence disease evolution. Therefore, the identification of specific mutations in this subgroup is mandatory to predict the clinical outcome and to select the most appropriate treatment approach. We found that molecular lesions in *TET2*, *SRSF2*, and *U2AF1* were associated with negative outcomes. Our data are in line with recent studies showing that *TET2* mutations and older age are independent prognostic factor in AML [23]. The *U2AF1* adverse prognostic impact on survival has been already reported in a limited AML cohort [24]. To the best of our knowledge, the data on the impact of *SRSF2* mutations on CR achievement were not previously reported in a cohort of patients with AML.

For the few patients (2%) with no evidence of DNA mutations, sequencing of a wider genome region, including regulatory and intronic sequences, and/or the use of an integrate analysis including other approach as comparative genomic hybridization arrays might identify rarer AML related genetic abnormalities and provide useful information for clinical decision making [25].

## 4. Patients and Methods

Out of 574 newly diagnosed AML patients enrolled into the NILG-AML 02/06 clinical trial, 270 subjects showed a normal karyotype. Molecular profile was performed on a total of 221 NK-AML with available diagnostic samples. Patients were affected by a de novo AML or by an AML secondary to chemo-radiotherapy or to a myelodysplastic/myeloproliferative syndrome (Table 1). This protocol was a randomized trial comparing ICE (idarubicin-cytarabine-etoposide) with sequential high dose (HD) chemotherapy in untreated patients with the intent to improve the early remission rate and to evaluate the impact on survival [12]. The trial protocol has been approved by the institutional review boards at each of the participating center (Comitato etico della provincia di Bergamo (CE150180), Comitato Etico Area Vasta Centro (CE150071), Comitato Etico città della salute e della scienza (CE150115), Comitato Etico Brianza (CE150179), Comitato Etico Interaziendale A.S.O. SS. Antonio e Biagio e C.Arrigo di Alessandria (CE150105), Comitato Etico di Brescia (CE150186), Comitato etico per la sperimentazione clinica - Comprensorio di Bolzano (CE150099), Comitato Etico Interaziendale Aso S.Croce E Carle (CE150123), CESC della Provincia di Venezia e IRCSS San Camillo(CE150073), Comitato Etico Val Padana (CE150177), Comitato Etico dell’IRCCS San Raffaele (CE150050), Comitato Etico Indipendente Istituto Clinico Humanitas (CE150081), Comitato Etico dell’Insubria (CE150185), Comitato Etico Indipendente della Fondazione IRCCS Istituto Nazionale dei Tumori di Milano (CE150053) and Comitato Etico Milano Area 2 (CE150176)).Informed consents for inclusion in the trial and for genetic analysis were obtained from all patients. Genomic DNA was isolated from mononuclear cells obtained from bone marrow or peripheral blood at diagnosis, containing at least 20% blasts. In the analysis of *FLT3*-ITD and D835 point mutations, *KTM2A-PTD*, *NPM1,* and *CEBPA* alterations were prospectively obtained with standard approaches (PCR analysis, enzymatic digestion, Sanger sequencing). In addition, we estimated the mutant to wild-type allelic ratio (AR) of *FLT3-ITD* using fragment length analysis technique [26]. Subsequently, on the same prospectively collected diagnostic samples, we obtained a more complete molecular profile by next generation sequencing (NGS) of targeted regions of a wide selection of myeloid neoplasms related genes. Two commercial NGS kits were applied to prepare DNA libraries for sequencing: Trusight Myeloid panel (Illumina, San Diego, CA, USA) and Sophia Myeloid Solution (SOPHiA GENETICS, SA, CH) investigating 54 and 30 gene regions, respectively (Table S1). The libraries were sequenced and demultiplexed on a MiSeq or MiniSeq instruments (Illumina, San Diego, CA). The median coverage was 6373 reads (range 44166–103) with 92% sequenced regions with > 500 and 87% with > 1000 reads. The limit of detection (LOD) for a reliable variant calling was down to 5% variant allele frequency (VAF), as recommended by both the producers. Frameshift and nonsense variants were always considered as relevant mutations. Single nucleotide variants were retained in the absence of description as genetic polymorphism into public databases of human polymorphisms (NCBI dbSNP (http://www.ncbi.nlm.nih.gov/snp; Build 137) and ExAC (http://exac.broadinstitute.org/)). Functional prediction for missense variants was derived from SIFT 1.03 (http://sift.jcvi.org) and PolyPhen2.0 (http://genetics.bwh.harvard.edu/pph2). For alterations of splicing sites and splicing related regions, we used the Human Splicing tool (Human Splicing Finder) to predict the effect on the splicing process. Finally, the description of other cancer specimens in terms of the identified mutations was checked against COSMIC database (http://cancer.sanger.ac.uk/cancergenome/projects/cosmic).

The clinical endpoints of the study were defined according to standard criteria [27]. Overall survival (OS) was defined as the probability of survival irrespective of disease state at any point in time from diagnosis. Patients alive at their last follow-up were censored. Disease free survival (DFS) was measured from the time of first CR until relapse or death. Baseline continuous characteristics were presented as median with range and compared using the Mann–Whitney U test. Categorical variables were reported with absolute and percentage frequencies and compared with Chi-squared test or Fisher’s exact test. OS and DFS were estimated by the Kaplan–Meier method and any differences were evaluated with a log-rank test. Cox models were used to estimate hazard ratios with 95% confidence intervals (CI) in univariate and multivariable analysis on survival outcomes. In this context, allogeneic hematologic stem cell transplantation (alloHSCT) was considered as a time-dependent event; Mantel–Byar tests and Simon–Makuch plots were used. In multivariable models, only factors with a *p* value < 0.2 in a corresponding univariate model were included. All reported *p* values are two-sided and a 5% significance level was set. All analyses were performed with R software, version 3.5.0.

## 5. Conclusions

In NK-AML, the accurate and in-depth molecular characterization did not lead to the recognition of a mutational profile associated with a different rate of response following an intensified induction chemotherapy program. No matter the induction chemotherapy, we identified mutations which are associated with different outcomes and which help to select the most appropriate consolidation strategies, namely alloHSCT. Finally, the identification of mutations that represent a potential treatment target for new drugs is now mandatory for offering patients new chemotherapy free therapeutic options.

## Figures and Tables

**Table 1:** Patients characteristics according to induction treatment.

Patients Characteristics and Mutations	All patients, *N* = 221	ICE, *N* = 117	sHD, *N =* 104	*p*
Median age, at diagnosis (range)	52.5 (19.8–74.8)	54.4 (23.6–74.8)	49.5 (19.8–72.2)	0.0324
≤60 years	166 (75.1)	81 (69.2)	85 (81.7)	0.0319
>60 years	55 (24.9)	36 (30.8)	19 (18.3)	
Sex				0.1765
Female	119 (53.8)	58 (49.6)	61 (58.7)	
Male	102 (46.2)	59 (50.4)	43 (41.3)	
AML category				0.0463
Non de novo	26 (11.8)	9 (7.7)	17 (16.3)	
De novo	195 (88.2)	108 (92.3)	87 (83.7)	
ECOG PS				0.4556
0-1	201 (91)	108 (92.3)	93 (89.4)	
2-3	20 (9)	9 (7.7)	11 (10.6)	
Hepatomegaly	17 (7.7)	8 (6.8)	9 (8.7)	0.6130
Splenomegaly	20 (9)	9 (7.7)	11 (10.6)	0.4556
Extramedullary involvement	34 (15.4)	16 (13.7)	18 (17.3)	0.4550
WBC count (×10^9^/L)				0.3677
≤50	155 (70.1)	79 (67.5)	76 (73.1)	
>50	66 (29.9)	38 (32.5)	28 (26.9)	
Hemoglobin (g/dL)	9.5 (4.3–14.1)	9.5 (5.1–14.1)	9.5 (4.3–13.9)	0.9144
Platelets(×10^9^/L)	59 (5–815)	64 (5–815)	57.5 (8–513)	0.8752
Bone marrow blast cells, %	80 (0–100)	83 (10–100)	80 (0–100)	0.4519
Peripheral blood blasts cells, %	52 (0–100)	50 (0–100)	55.5 (0–100)	0.6909
Consolidation				0.3276
No alloHSCT	119 (67.9)	60 (59.4)	59 (66.3)	
alloHSCT	71 (32.1)	41 (40.6)	30 (33.7)	
*FLT3* wt., *NPM1* wt	90/216 (41.7)	42/112 (37.5)	48/104 (46.2)	0.1974
*FLT3-ITD* low ratio, *NPM1* wt	6/221 (2.7)	6/117 (5.1)	0/104 (0)	0.0307
*FLT3-ITD* high ratio, *NPM1* wt	8/221 (3.6)	4/117 (3.4)	4/104 (3.8)	1.0000
*FLT3* wt, *NPM1 +*	66/221 (29.9)	34/117 (29.1)	32/104 (30.8)	0.7817
*FLT3-ITD* low ratio, *NPM1 +*	15/221 (6.8)	10/117 (8.5)	5/104 (4.8)	0.2700
*FLT3-ITD* high ratio, *NPM1 +*	*31/221 (14)*	*16/117 (13.7)*	*15/104 (14.4)*	*0.8730*

wt, wild type; ICE, standard idarubicin-cytarabine-etoposide chemotherapy; sHD, sequential high dose chemotherapy.

**Table 2:** Univariate analysis on patients outcome.

Patients Characteristics	CR		OS		DFS	
	HR	*p*	HR	*p*	HR	*p*
HDS	0.94 (0.44–2.03)	0.8731	0.86 (0.59–1.26)	0.4318	0.82 (0.54–1.23)	0.3276
HSCT	-	-	0.31 (0.18–0.51)	0.0000	0.29 (0.17–0.48)	<0.0001
Age > 60	0.21 (0.09–0.45)	0.0001	2.67 (1.81–3.95)	0.0000	1.92 (1.22–3.02)	0.0047
Sex male	1.67 (0.77–3.79)	0.2019	0.95 (0.65–1.39)	0.7982	1.06 (0.7–1.58)	0.7901
De novo	2.62 (0.94–6.69)	0.0503	0.73 (0.42–1.27)	0.2665	0.76 (0.41–1.43)	0.3975
ECOG PS 2–3	0.25 (0.09–0.73)	0.0076	2.24 (1.25–4.01)	0.0065	1.06 (0.46–2.43)	0.886
WBC count > 50	0.74 (0.34–1.7)	0.4621	1.61 (1.09–2.39)	0.0179	1.37 (0.89–2.12)	0.1533
***NPM1***	1.76 (0.82–3.92)	0.1541	0.71 (0.48–1.04)	0.0780	0.76 (0.51–1.14)	0.1864
VAF ≤ 0.4	2.18 (0.87–6.27)	0.1162	0.67 (0.42–1.05)	0.0785	0.89 (0.57–1.39)	0.6075
VAF > 0.4	1.3 (0.5–3.81)	0.6031	0.8 (0.48–1.33)	0.3924	0.59 (0.33–1.08)	0.0866
***FLT3***-ITD	0.79 (0.34–1.93)	0.5811	2.23 (1.5–3.32)	0.0001	2.18 (1.43–3.33)	0.0003
***FLT3***-ITD low	0.95 (0.29–4.29)	0.9380	1.67 (0.9–3.08)	0.1032	1.55 (0.8–3.04)	0.1966
***FLT3***-ITD high	0.87 (0.34–2.51)	0.7813	2.43 (1.56–3.78)	0.0001	2.6 (1.62–4.18)	0.0001
***DNMT3A***	1.01 (0.47–2.25)	0.9799	1.25 (0.85–1.83)	0.2606	1.49 (0.99–2.23)	0.0553
***TET2***	0.23 (0.1–0.54)	0.0006	1.38 (0.85–2.24)	0.1926	0.94 (0.5–1.76)	0.8357
***RUNX1***	0.42 (0.17–1.08)	0.0590	2.25 (1.43–3.55)	0.0005	1.95 (1.15–3.3)	0.0132
***IDH2***	0.68 (0.25–2.17)	0.4754	0.77 (0.4–1.47)	0.4247	1.05 (0.56–1.97)	0.8732
CEBPA2 *	3.37 (0.66–61.69)	0.2450	0.26 (0.1–0.71)	0.0088	0.21 (0.06–0.65)	0.007
***FLT3_***PM	1.57 (0.42–10.17)	0.5605	0.45 (0.2–1.04)	0.0608	0.38 (0.16–0.94)	0.0371
***IDH1***	0.98 (0.3–4.36)	0.9714	0.95 (0.51–1.78)	0.8781	0.98 (0.51–1.89)	0.9575
***ASXL1***	0.25 (0.09–0.73)	0.0076	1.54 (0.86–2.76)	0.1434	1.3 (0.63–2.68)	0.4827
CEBPA1 *	2.32 (0.44–42.85)	0.4243	0.56 (0.23–1.39)	0.2133	0.63 (0.25–1.55)	0.3141
***PTPN11***	2.95 (0.57–54.09)	0.3022	0.46 (0.19–1.13)	0.0908	0.58 (0.26–1.33)	0.2018
***SRSF2***	0.12 (0.04–0.34)	0.0001	1.43 (0.77–2.67)	0.2596	0.8 (0.29–2.17)	0.6553
***NRAS***	0.74 (0.22–3.37)	0.6557	1.46 (0.78–2.72)	0.2387	1.31 (0.64–2.71)	0.4607
***KMT2A-***PTD	2.42 (0.46–44.57)	0.4027	1.34 (0.67–2.67)	0.4037	1.44 (0.72–2.88)	0.298
***STAG2***	0.35 (0.1–1.36)	0.0990	1.33 (0.61–2.88)	0.4712	1.3 (0.52–3.23)	0.5691
***BCOR***	1.95 (0.36–36.43)	0.5310	1.58 (0.73–3.44)	0.2456	1.52 (0.66–3.51)	0.3258
***KRAS***	1.84 (0.34–34.31)	0.5648	0.81 (0.33–2)	0.6546	1.1 (0.48–2.52)	0.8178
***WT1***	1.84 (0.34–34.31)	0.5648	0.8 (0.32–1.95)	0.6185	0.75 (0.28–2.04)	0.5736
***GATA2***	>99.99 (0–NA)	0.9894	0 (0–Inf)	0.9953	0 (0–Inf)	0.9954
***SF3B1***	1.32 (0.23–24.91)	0.7976	1.42 (0.66–3.06)	0.3689	1.8 (0.83–3.9)	0.1348
***U2AF1***	1.32 (0.23–24.91)	0.7976	2.69 (1.3–5.55)	0.0075	3.57 (1.64–7.74)	0.0013
***FLT3***wt, ***NPM1*** wt	0.58 (0.26–1.28)	0.1744	1.12 (0.76–1.65)	0.5682	1.08 (0.71–1.63)	0.726
***FLT3***-ITD low ratio, ***NPM1*** wt	0.81 (0.12–15.82)	0.8505	1.55 (0.57–4.23)	0.388	1.3 (0.41–4.1)	0.6572
***FLT3***-ITD high ratio, ***NPM1*** wt	>99.99 (0–NA)	0.9861	2.09 (0.97–4.49)	0.0602	2.47 (1.14–5.34)	0.0214
***FLT3***wt, ***NPM1*** +	3.27 (1.21–11.42)	0.0336	0.35 (0.21–0.59)	0.0001	0.44 (0.27–0.71)	0.0009
***FLT3***-ITD low ratio, ***NPM1*** +	1.06 (0.28–7.03)	0.9361	1.27 (0.62–2.62)	0.5089	1.25 (0.58–2.7)	0.5683
***FLT3***-ITD high ratio, ***NPM1*** +	0.63 (0.25–1.83)	0.3602	2.15 (1.33–3.47)	0.0017	2.21 (1.31–3.75)	0.0031

* CEBPA2 and *CEBPA*1 indicate the presence of double or single mutation, respectively.

**Table 3:** Multivariable analysis for patients characteristics, treatments and mutations in the complete cohort.

Patients Characteristics	CR		OS		DFS	
	HR	*p*	HR	*p*	HR	*p*
HSCT	-	-	0.34 (0.19–0.60)	0.0002	0.34 (0.19–0.60)	<0.0001
Age > 60	0.43 (0.15–1.22)	0.1049	1.37 (0.78–2.40)	0.2661	0.89 (0.51–1.55)	0.6864
De novo	2.17 (0.55–7.76)	0.2460	-	-	-	-
ECOG PS 2–3	0.26 (0.07–1)	0.0398	1.09 (0.42–2.85)	0.8559	-	-
WBC count > 50	-	-	1.20 (0.67–2.14)	0.5456	1.07 (0.63–1.82)	0.8023
***NPM1***	1.06 (0.33–3.3)	0.9239	0.58 (0.32–1.06)	0.0761	0.49 (0.28–0.88)	0.0163
***FLT3-ITD***	-	-	2.76 (1.56–4.91)	0.0005	2.81 (1.66–4.73)	0.0001
***DNMT3A***	-	-	-	-	1.62 (0.95–2.77)	0.0772
***TET2***	0.4 (0.13–1.24)	0.1048	0.74 (0.33–1.66)	0.4715	-	-
***RUNX1***	0.54 (0.15–2.03)	0.3501	1.25 (0.60–2.61)	0.5567	0.89 (0.43–1.87)	0.7638
CEBPA2 *	-	-	0.20 (0.06–0.68)	0.0097	0.17 (0.05–0.57)	0.0040
***FLT3_PM***	-	-	0.65 (0.23–1.89)	0.4325	0.65 (0.25–1.67)	0.3731
***ASXL1***	0.87 (0.19–4.63)	0.8628	0.42 (0.16–1.08)	0.0713	-	-
***PTPN11***	-	-	0.60 (0.21–1.73)	0.3450	-	-
***SRSF2***	0.24 (0.06–0.95)	0.0376	-	-	-	-
***STAG2***	0.97 (0.18–6.65)	0.9715	-	-	-	-
***SF3B1***	-	-	-	-	1.02 (0.42–2.45)	0.9663
***U2AF1***	-	-	4.19 (1.72–10.23)	0.0016	5.54 (2.25–13.66)	0.0002

* *CEBPA*2 indicates the presence of a double mutation.

**Table 4:** Univariate analysis for patients characteristics, treatments and mutations in patients lacking both *FLT3*-ITD and *NPM1* alterations (*n* = 90 and *n* = 75 achieving CR).

Patients Characteristics	CR *n* = 90		OS *n* = 90		DFS *n* = 75	
	HR	*p*	HR	*p*	HR	*p*
HDS	0.51 (0.15–1.59)	0.2622	1.29 (0.72–2.32)	0.3877	1.05 (0.56–1.98)	0.8837
HSCT	-	-	0.43 (0.21–0.89)	0.0229	0.42 (0.2–0.88)	0.0225
Age > 60	0.51 (0.16–1.69)	0.2524	2.28 (1.26–4.15)	0.0068	2.63 (1.35–5.11)	0.0043
Sex male	2.38 (0.78–7.77)	0.1336	0.63 (0.35–1.13)	0.1194	0.86 (0.45–1.64)	0.6537
De novo	2.38 (0.65–7.98)	0.1657	0.63 (0.33–1.22)	0.1729	0.58 (0.27–1.23)	0.1569
ECOG PS 2–3	1 (0.15–19.93)	1.0000	2.66 (0.95–7.47)	0.0635	2 (0.61–6.53)	0.2504
WBC count > 50	1.22 (0.19–23.93)	0.8605	0.54 (0.13–2.24)	0.3955	0.28 (0.04–2.03)	0.2066
***DNMT3A***	0.48 (0.12–2.41)	0.3219	2.77 (1.32–5.8)	0.0068	3.83 (1.67–8.76)	0.0015
***TET2***	0.21 (0.05–0.81)	0.0195	2.26 (1.09–4.71)	0.0286	2.33 (0.9–5.99)	0.0797
***RUNX1***	0.41 (0.13–1.37)	0.1323	2.36 (1.28–4.35)	0.0060	1.96 (0.97–3.96)	0.0608
***IDH2***	0.52 (0.15–2.13)	0.3297	1.03 (0.46–2.3)	0.9439	1.56 (0.68–3.54)	0.2909
CEBPA2 *	4.5 (0.81–84.34)	0.1598	0.16 (0.05–0.53)	0.0026	0.12 (0.03–0.5)	0.0035
***ASXL1***	0.27 (0.08–1.03)	0.0466	1.26 (0.59–2.7)	0.5564	1.12 (0.44–2.86)	0.8194
CEBPA1 *	>99.99 (0–NA)	0.9934	0.45 (0.14–1.45)	0.1801	0.72 (0.25–2.03)	0.5315
***SRSF2***	0.08 (0.02–0.31)	0.0003	1.33 (0.59–2.98)	0.4889	0.7 (0.17–2.93)	0.6305
***NRAS***	0.57 (0.11–4.14)	0.5123	2.88 (1.28–6.48)	0.0105	2.9 (1.13–7.48)	0.0272
***KMT2A-***PTD	3.5 (0.62–65.89)	0.2437	1.05 (0.49–2.26)	0.8960	1.22 (0.56–2.66)	0.6168
***STAG2***	0.79 (0.17–5.67)	0.7783	1.16 (0.45–2.98)	0.7603	1.57 (0.6–4.09)	0.3593
***BCOR***	>99.99 (0–NA)	0.9908	1.31 (0.46–3.71)	0.6128	1.36 (0.48–3.9)	0.5639
***KRAS***	>99.99 (0–NA)	0.9914	0.61 (0.08–4.44)	0.6276	0.6 (0.08–4.37)	0.6138
***WT1***	>99.99 (0–NA)	0.9903	0.58 (0.18–1.86)	0.3584	0.6 (0.18–1.96)	0.3983
***GATA2***	>99.99 (0–NA)	0.9937	0 (0–Inf)	0.9973	0 (0–99.99)	0.9973
***SF3B1***	1.44 (0.23–28.05)	0.7417	1.4 (0.59–3.3)	0.4479	1.77 (0.74–4.26)	0.2010
***U2AF1***	1.67 (0.27–32.28)	0.6406	3.03 (1.4–6.58)	0.0049	3.89 (1.69–8.93)	0.0014

* CEBPA2 and *CEBPA*1 indicate the presence of double or single mutation, respectively.

**Table 5:** Multivariate analysis for patients characteristics, treatments and mutations in *FLT3* wt and *NPM1* wt patients.

Patients Characteristics	CR		OS		DFS	
	HR (95% CI)	*p*	HR (95% CI)	*p*	HR (95% CI)	*p*
HSCT	-	-	0.42 (0.16–1.09)	0.0744	0.24 (0.09–0.62)	0.0032
Age > 60	-	-	1.02 (0.43–2.41)	0.9624	1.12 (0.51–2.45)	0.7778
Sex male	2.57 (0.66–11.53)	0.1846	0.77 (0.33–1.78)	0.5462	-	-
De novo	0.65 (0.11–2.96)	0.5985	0.88 (0.36–2.16)	0.7753	1.29 (0.50–3.33)	0.5997
ECOG PS 2–3	-	-	2.24 (0.54–9.31)	0.2681	-	-
***DNMT3A***	-	-	2.58 (0.80–8.28)	0.1105	3.57 (1.07–11.89)	0.0383
***TET2***	0.15 (0.02–0.94)	0.0387	2.32 (0.70–7.63)	0.1670	1.93 (0.62–6.03)	0.2600
***RUNX1***	0.44 (0.1–1.99)	0.2747	2.20 (0.93–5.24)	0.0741	1.93 (0.83–4.50)	0.1277
CEBPA2 *	3.93 (0.47–98.06)	0.2806	0.20 (0.04–0.92)	0.0387	0.13 (0.03–0.58)	0.0070
***ASXL1***	1.38 (0.24–11.14)	0.7367	-	-	-	-
***NRAS***	-	-	1.21 (0.38–3.87)	0.7457	1.05 (0.34–3.29)	0.9284
***SRSF2***	0.08 (0.01–0.5)	0.0093	-	-	-	-
***U2AF1***	-	-	3.39 (1.16–9.92)	0.0260	3.81 (1.35–10.78)	0.0117

* CEBPA2 indicates the presence of a double mutation.

## Supplementary Materials

The following are available online at https://www.mdpi.com/2072-6694/12/8/2242/s1, Figure S1: Forest plot of induction treatment, Figure S2: Kaplan-Meier curves of Overall Survival and Disease-free Survival according to *FLT3*-ITD and *NPM1* mutations, Figure S3: Kaplan-Meier curves of Overall Survival (OS) in different induction treatments, Table S1: Gene sequenced using Trusight Myeloid panel (Illumina, San Diego, CA, USA) and Sophia Myeloid Solution (SOPHiA GENETICS, SA, CH) (indicated with *).
