# Supplementary material for: High Throughput Molecular Characterization of Normal Karyotype Acute Myeloid Leukemia in the Context of the Prospective Trial 02/06 of the Northern Italy Leukemia Group (NILG)
Source: Cancers (Basel). 2020 Aug 11;12(8):2242. doi: 10.3390/cancers12082242 (PMC7464263; doi:10.3390/cancers12082242)
Supplement: Supplementary file 1 [file cancers-12-02242-s001.pdf]

Supplementary Materials

High Throughput Molecular Characterization of Normal Karyotype Acute Myeloid Leukemia in the Context of The Prospective Trial 02/06 of the Northern Italy Leukemia Group (NILG)

Silvia Salmoiraghi, Roberta Cavagna, Pamela Zanghì, Chiara Pavoni, Anna Michelato, Ksenija Buklijas, Lara Elidi, Tamara Intermesoli, Federico Lussana, Elena Oldani, Chiara Caprioli, Paola Stefanoni, Giacomo Gianfaldoni, Ernesta Audisio, Elisabetta Terruzzi, Lorella De Paoli, Erika Borlenghi, Irene Cavattoni, Daniele Mattei, Annamaria Scattolin, Monica Tajana, Fabio Ciceri, Elisabetta Todisco, Leonardo Campiotti, Paolo Corradini, Nicola Fracchiolla, Renato Bassan, Alessandro Rambaldi and And Orietta Spinelli

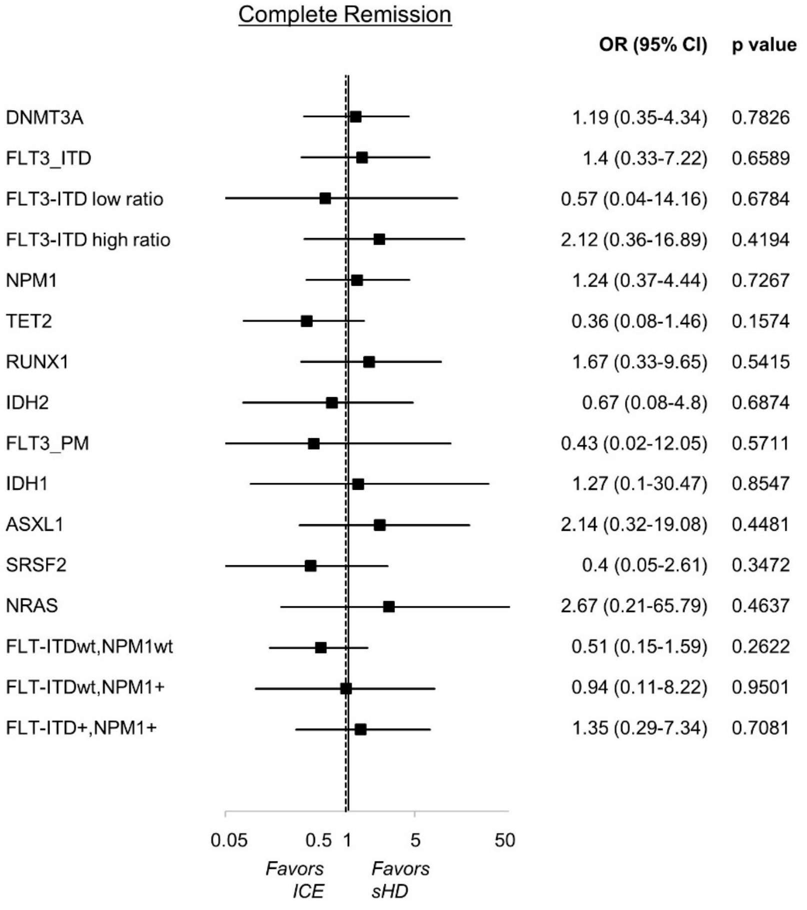

Figure S1. Forest plot of induction treatment. Effects of treatments on CR, according to the gene molecular alteration detected in our cohort of patients.

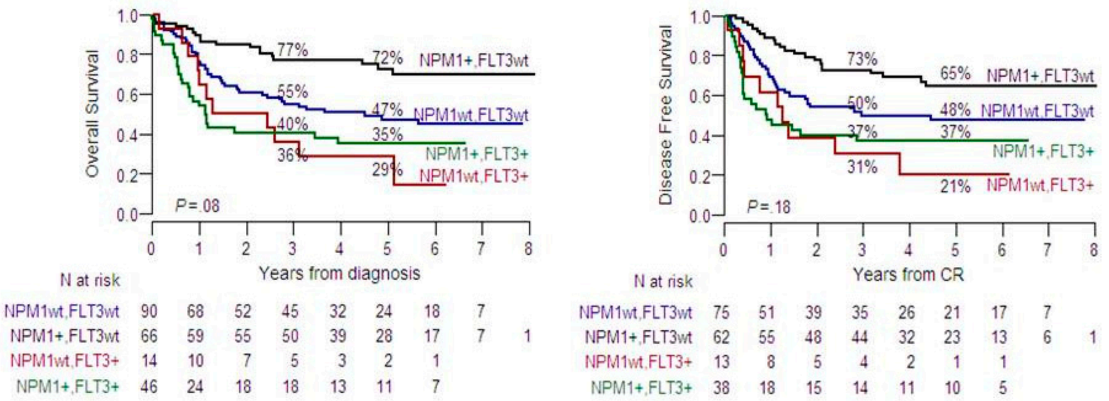

Figure S2. Kaplan-Meier curves of Overall Survival and Disease-free Survival according to *FLT3*-ITD and *NPM1* mutations. 3-year and 5-year estimates and global *P* values are reported.

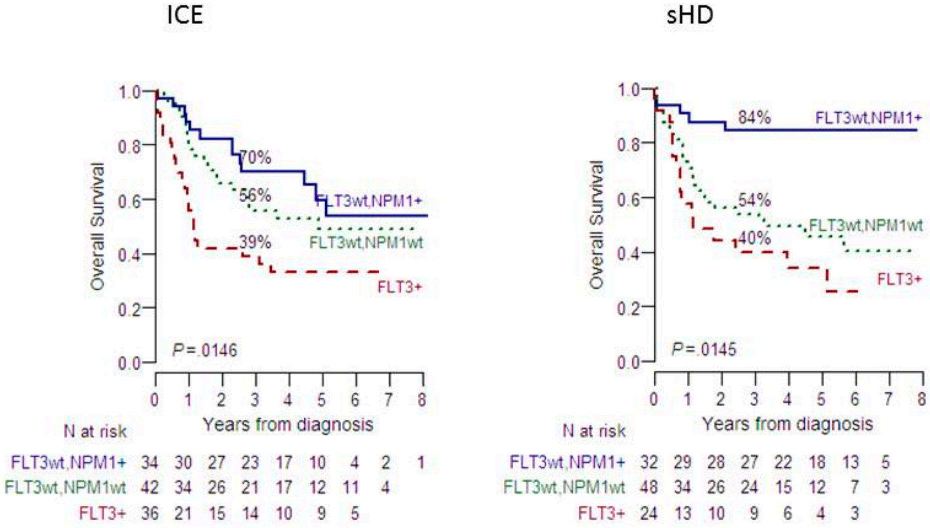

Figure S3. Kaplan-Meier curves of Overall Survival (OS) in different induction treatments, according to *FLT3*-ITD and *NPM1* mutations. 3-year OS estimates and global *P* values are reported.

Table S1. Gene sequenced using Trusight Myeloid panel (Illumina, San Diego, CA, USA) and Sophia Myeloid Solution (SOPHiA GENETICS, SA, CH) (indicated with *).

Gene	Target Region (Exons)	Gene	Target Region (Exons)	Gene	Target Region (Exons)	Gene	Target Region (Exons)
*ABL1**	4-6	*DNMT3A**	Full	*KDM6A*	Full	*RAD21*	Full
*ASXL1**	12	*ETV6**	Full	*KIT**	2,8–11,13,17	*RUNX1**	Full
ATRX	8–10,17–31	*EZH2**	Full	*KRAS**	2,3	*SETBP1**	4 (partial)
BCOR	Full	*FBXW7*	9–11	*MLL*	5-8	*SF3B1**	13–16
BCORL1	Full	*FLT3**	14–15,20	*MPL**	10	*SMC1A*	2,11,16–17
*BRAF**	15	*GATA1*	2	*MYD88*	3–5	*SMC3*	10,13,19,23,25,28
*CALR**	9	*GATA2*	2-6	*NOTCH1*	26–28,34	*SRSF2**	1
*CBL**	8,9	*GNAS*	8,9	*NPM1**	12	*STAG2*	Full
CBLB	9,10	*HRAS**	2,3	*NRAS**	2,3	*TET2**	3–11
CBLC	9,10	*IDH1**	4	*PDGFRA*	12,14,18	*TP53**	2–11
CDKN2A	Full	*IDH2**	4	*PHF6*	Full	*U2AF1**	2,6
*CEBPA**	Full	*IKZF1*	Full	*PTEN*	5, 7	*WT1**	7,9
*CSF3R**	14–17	*JAK2**	12,14	*PTPN11*	3, 13	*ZRSR2**	Full
CUX1	Full	*JAK3*	13				
